# Benefits and unintended consequences of antimicrobial de-escalation: Implications for stewardship programs

**DOI:** 10.1371/journal.pone.0171218

**Published:** 2017-02-09

**Authors:** Josie Hughes, Xi Huo, Lindsey Falk, Amy Hurford, Kunquan Lan, Bryan Coburn, Andrew Morris, Jianhong Wu

**Affiliations:** 1 Centre for Disease Modelling, York University, Toronto, Ontario, Canada; 2 Department of Mathematics, Ryerson University, Toronto, Ontario, Canada; 3 Dalla Lana School of Public Health, University of Toronto, Toronto, Ontario, Canada; 4 Department of Biology and Department of Mathematics and Statistics, Memorial University of Newfoundland, St. John’s, Newfoundland, Canada; 5 Department of Laboratory Medicine & Pathobiology, University of Toronto, Toronto, Ontario, Canada; 6 Department of Medicine, Sinai Health System & University Health Network, Toronto, Ontario, Canada; 7 Department of Medicine, University of Toronto, Toronto, Ontario, Canada; Emory University School of Medicine, UNITED STATES

## Abstract

Sequential antimicrobial de-escalation aims to minimize resistance to high-value broad-spectrum empiric antimicrobials by switching to alternative drugs when testing confirms susceptibility. Though widely practiced, the effects de-escalation are not well understood. Definitions of interventions and outcomes differ among studies. We use mathematical models of the transmission and evolution of *Pseudomonas aeruginosa* in an intensive care unit to assess the effect of de-escalation on a broad range of outcomes, and clarify expectations. In these models, de-escalation reduces the use of high-value drugs and preserves the effectiveness of empiric therapy, while also selecting for multidrug-resistant strains and leaving patients vulnerable to colonization and superinfection. The net effect of de-escalation in our models is to increase infection prevalence while also increasing the probability of effective treatment. Changes in mortality are small, and can be either positive or negative. The clinical significance of small changes in outcomes such as infection prevalence and death may exceed more easily detectable changes in drug use and resistance. Integrating harms and benefits into ranked outcomes for each patient may provide a way forward in the analysis of these tradeoffs. Our models provide a conceptual framework for the collection and interpretation of evidence needed to inform antimicrobial stewardship.

## Introduction

Strategies that reduce the costs associated with antimicrobial use while maintaining the benefits are urgently needed. Antimicrobial de-escalation aims to reduce costs by switching from antimicrobials that provide good empiric coverage to alternatives based on laboratory susceptibility results, stopping unecessary or redundant treatment, and switching from IV to oral therapy [1, 2]. Here, we consider the implications of sequential switching from empiric to alternative drugs, hereafter referred to as de-escalation. Though commonly practiced, the effects of de-escalation are not well understood [1, 3, 4]. De-escalation is associated with lower mortality in observational studies, but this relationship may be confounded by other determinants of improvement [3]. Non-inferiority trials have not found a change in mortality [5, 6], and have not been designed to assess effects of de-escalation on resistance [3]. Unintended increases in the emergence of multidrug-resistant (MDR) organisms [6] and superinfections [5] have been observed following antimicrobial de-escalation. Definitions of interventions and outcomes differ among studies, making comparison difficult [3].

To inform the design of future studies we develop a mathematical model of the ecological and evolutionary consequences of antimicrobial de-escalation in an intensive care unit (ICU). Existing models have informed our understanding of other stewardship and infection control interventions [7–14] but this is the first theoretical examination of de-escalation. We initially focus on the evolution and transmission of *Pseudomonas aeruginosa* in the ICU, then consider the wider implications of our results. In general, de-escalation differs among pathogens, infection types, patient groups, and treatment contexts, and the details matter. *P. aeruginosa* is primarily a nosocomial pathogen [15], and is particularly adept at developing resistance [16–18]. Thus, the potential benefits of antimicrobial stewardship are high for this organism.

## Materials and methods

### Modeling the effects of antimicrobial de-escalation

We model the effects of antimicrobial de-escalation on *P. aeruginosa* transmission, infection, and evolutionary dynamics in an intensive care unit, and consequences for patients. Specifically, we consider the effects of three therapy adjustment strategies on the evolution of *P. aeruginosa* resistance to piperacillin-tazobactam, which is often used for empiric therapy of severe infections in the ICU because it provides good coverage of common pathogens [19–21], and ciprofloxacin which is often used for definitive treatment of susceptible *P. aeruginosa* infections [22–27]. Ciprofloxacin is the preferred alternative because it provides poorer empiric coverage of common Gram-positive pathogens such as *Staphylococcus aureus* [21], and can be given orally. We focus on the effects of drug switching, and do not consider drug combinations.

**Fig 1:**
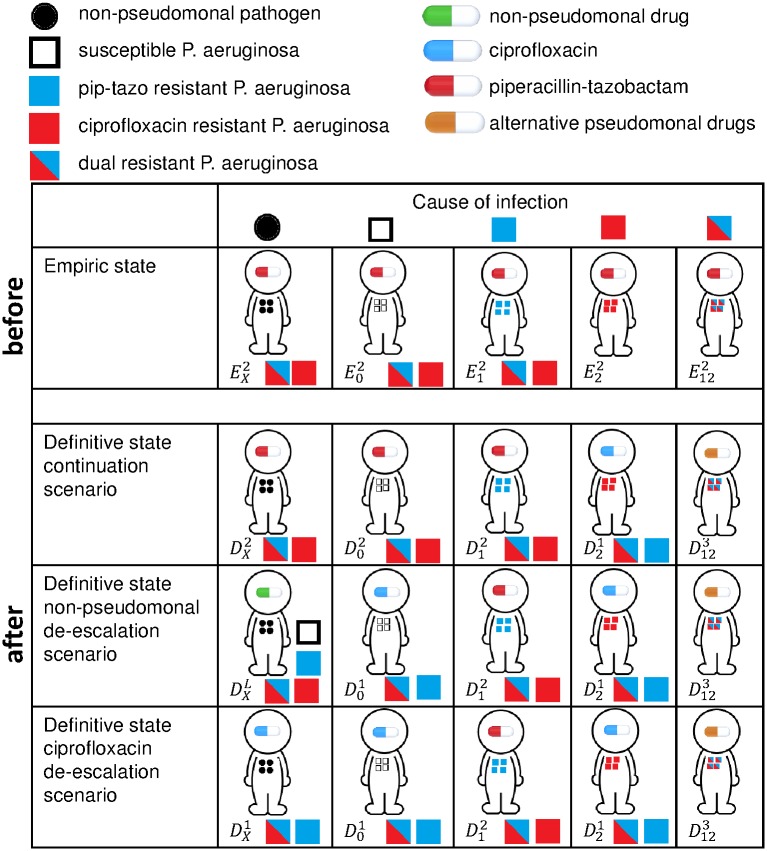
Direct effects of antimicrobial de-escalation. Infections can be caused by resistant or susceptible *P. aeruginosa* strains, or non-pseudomonal pathogens (circles and squares inside people, columns). Strains that are treated (pills inside people) effectively can be replaced by resistant strains (circles and squares outside people). All infections are treated empirically with piperacillin-tazobactam (row 1). In the continuation scenario, susceptible patients are also treated definitively with piperacillin-tazobactam (row 2). In the non-pseudomonal de-escalation scenario, patients with non-pseudomonal infections are treated definitively with non-pseudomonal drugs, while susceptible *P. aeruginosa* infections are treated with ciprofloxacin (row 3). In the ciprofloxacin scenario, both non-pseudomonal and susceptible *P. aeruginosa* infections are treated definitively with ciprofloxacin (row 4).

Infected patients are treated empirically with piperacillin-tazobactam until testing confirms the identity and susceptibilities of the infecting pathogen (row 1 of Fig 1). In the non-pseudomonal de-escalation scenario, patients confirmed to have a non-pseudomonal infection are switched to a non-pseudomonal drug, while patients with susceptible *P. aeruginosa* infections are switched to ciprofloxacin (row 3 of Fig 1). In the continuation scenario, alternative drugs are only given when testing confirms resistance to piperacillin-tazobactam (row 2 of Fig 1). In the ciprofloxacin de-escalation scenario, ciprofloxacin is used to treat all confirmed susceptible infections (row 4 row of Fig 1). The ciprofloxacin scenario is unrealistic, but provides insight into model behaviour. Infections resistant to both piperacillin-tazobactam and ciprofloxacin are treated with a last-resort drug such as a carbapenem or aminoglycoside (5th column of Fig 1).

Both de-escalation strategies (rows 3 and 4 of Fig 1) reduce the use of piperacillin-tazobactam. This should reduce selection for resistance to piperacillin-tazobactam, thus preserving the effectiveness of empiric therapy. The use of ciprofloxacin and alternative drugs will increase with de-escalation, but resistance to definitive drugs is less problematic than resistance to empiric therapy because definitive therapy is informed by laboratory susceptibility testing. De-escalation also leaves patients vulnerable to colonization and subsequent infection by *P. aeruginosa* strains that are susceptible to piperacillin-tazobactam but unaffected by ciprofloxacin (rows 3 and 4 of Fig 1) or non-pseudomonal alternatives (row 3 of Fig 1). We use mathematical models to better understand the implications of these tradeoffs.

Mathematical models always represent a compromise between realism and simplicity. To minimize complexity, we focus on the evolution of *P. aeruginosa* resistance to piperacillin-tazobactam and ciprofloxacin, ignoring resistance to alternative non-pseudomonal and last-resort drugs. Thus, we have not included all the possible costs of antimicrobial de-escalation. We have omitted MDR resistance mechanisms such as efflux pumps [28–32], so the models represent a best-case scenario for the preservation of empiric therapy. Finally, we have omitted *Clostridium difficile* infection dynamics [33–36]. Thus, our models are sufficiently complex to demonstrate some possible tradeoffs, but not to determine whether, and in what contexts, de-escalation is good policy.

#### Patient categorization and notation

Patients are first categorized by their infection status: (*U*) uninfected; (*E*) infected, receiving empiric therapy, and expecting an accurate diagnosis; (*F*) infected, receiving empiric therapy, and expecting an inaccurate diagnosis because they have developed resistance during therapy; (*D*) infected and receiving definitive therapy; or (*S*) superinfected by *P. aeruginosa*. Infections can be caused by *P. aeruginosa* or other pathogens that cause similar symptoms (Fig 1).

**Fig 2:**
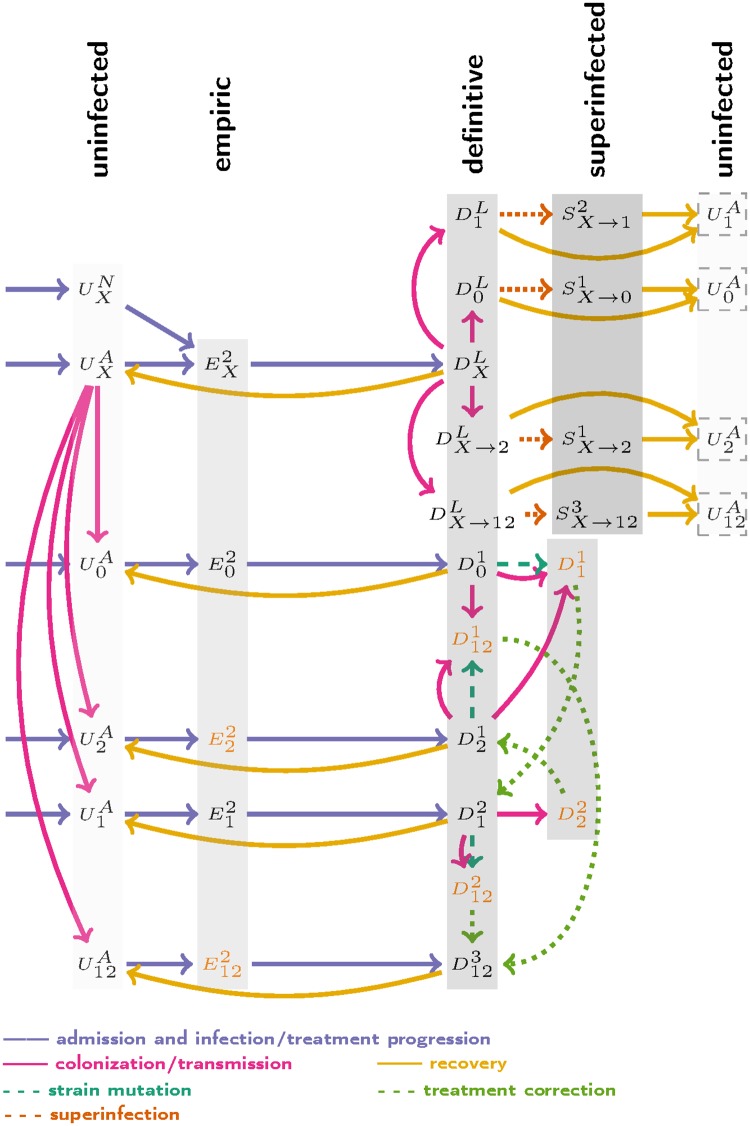
Simplified flow diagram of non-pseudomonal de-escalation. Subscripts denote the infecting or colonizing strain and superscripts denote the treatment status. See “Patient categorization and notation” for details. Orange text indicates inadequate treatment. Patients experiencing strain conversions during empiric therapy, discharge processes and death processes are not included in the simplified flow diagram. See S1–S3 Figs for complete dynamics.

A subscript denotes the *P.aeruginosa* strain carried by a patient, if any (Fig 1): (_*X*_) is a non-pseudomonal pathogen, (_1_) is a *P.aeruginosa* strain resistant to ciprofloxacin, (_2_) is strain resistant to piperacillin-tazobactam, (_12_) is a strain resistant to both drugs, and (_0_) is a strain susceptible to both drugs. Patients initially infected by species _*X*_ and subsequently colonized by *P.aeruginosa* can develop superinfections; we mark these patients as (_*X* → *i*_) with *i* = 0, 1, 2, 12 referring to different *P.aeruginosa* strains (Fig 2).

Finally, the treatment status of patients is noted in a superscript (Fig 1): (^1^) ciprofloxacin indicates ciprofloxacin treatment, (^2^), indicates pipercillin-tazobactam treatment, (^*L*^) indicates treatment with an alternative drug for non-pseudomonal infections, and (^3^) indicates treatment for dual-resistant infections, (^*A*^) indicates prior drug exposure, and (^*N*^) indicates no prior drug exposure.

Fig 2 summarizes model dynamics in non-pseudomonal de-escalation scenario. Complete descriptions of the three treatment scenarios, with equations, can be found in S1 Appendix. Model parameters are summarized in Table 1. We assume large ranges for uncertain parameters, giving confidence that the full range of possible outcomes has been explored at the cost of oversampling unlikely outcomes.

**Table 1:** De-escalation model parameters.

Symbol	Value	Definition
Parameters with fixed values		
*N*	16	Number of patients in ICU
*a*	0.6	Fraction of patients admitted with prior exposure to antimicrobials [13]
*τ*	1/3 day^−1^	Rate of finishing empiric therapy
*τ*_1_	1/5 ∼ 1/3 day^−1^	Rate of correcting failed definitive treatment
*τ*_2_	1/4 day^−1^	Rate of finishing an effective definitive treatment
Parameters with clear ranges		
*m*	0 ∼ 0.1	Fraction of patients admitted colonized [13]
*σ*_*x*_	0.013 ∼ 0.0203 day^−1^	Infection rate of patients colonized by other species [37]
*σ*_*c*_	0.05 ∼ 0.14 day^−1^	Infection rate of patients colonized by *P.aeruginosa* [13, 15]
*τ*_3_	$\frac{1}{15}∼\frac{1}{4}$ day^−1^	Rate of finishing an effective treatment to superinfection [38]
*κ*_*μ*_	0.49 ∼ 1.0	Hazard ratio of discharge with nosocomial infection [39, 40]
*κ*_*ν*_	1.0 ∼ 2.3	Hazard ratio of death with nosocomial infection [39, 40]
*δ*	4%∼40%	Difference in probability of death between effective and ineffective empiric therapy after 10 days [41, 42]
Uncertain parameters with large ranges		
*β*	0.01 ∼ 1 day^−1^	Transmission rate
*r*_1_	0 ∼ 0.7	Fraction of patients admitted colonized with strain 1
*r*_2_	0 ∼ *r*_1_	Fraction of patients admitted colonized with strain 2
*ε*_1_	0 ∼ 0.03 day^−1^	Rate of emergence of ciprofloxacin resistance
*ε*_2_	0 ∼ 0.03 day^−1^	Rate of emergence of piperacillin-tazobactam resistance
*μ*	0.025 ∼ 0.5 day^−1^	Discharge rate of patients without nosocomial infection
*ν*	0.005 ∼ 0.05 day^−1^	Death rate of patients without nosocomial infection
*η*	0 ∼ 100%	Probability of emergence of superinfection
*ϑ*	0 ∼ 100%	Hazard ratio of finishing an effective treatment to multi-drug resistant strain infection

#### Colonization, infection, and the emergence of resistance

We distinguish infected patients and patients that are colonized by *P. aeruginosa* but do not have symptoms of a *P. aeruginosa* infection [9, 13]. Patients remain *P. aeruginosa* carriers for 12-25 days [43], which is longer than the normal stay in the ICU [5, 44–46], so we assume that patients remain colonized for the duration of their stay [43].

Patients with a history of antimicrobial exposure are at higher risk of *P. aeruginosa* colonization [15, 47, 48]. For simplicity, we assume only exposed patients are colonized [13]. Patients with previous antimicrobial exposure can be colonized by resistant or susceptible *P. aeruginosa* strains, and patients receiving treatment can only be colonized by a strain that is resistant to that treatment. Transmission of *P.aeruginosa* strains among exposed patients is modeled by mass action with a transmission rate *β* [7, 49], without distinguishing among trasmission pathways [47, 50] or bacterial loads [51]. We examine sensitivity to a broad range of *β* (0.01 to 1 per day).

Colonized patients develop *P. aeruginosa* infections at a rate *σ*_*c*_ ∈ [0.05, 0.14] [13, 15]. Uncolonized patients develop infections with similar symptoms caused by other pathogens at a lower rate *σ*_*x*_ ∈ [0.013, 0.0203] [37]. See S1 Appendix for *σ*_*c*_ and *σ*_*x*_ estimation methods. Patients that become colonized while being treated for a non-pseudomonal infection develop *P. aeruginosa* superinfections with a probability *η*, which varies broadly between 0 and 100%.

The models allow resistant strains to replace dominant sensitive strains given antimicrobial selection pressure [7]. Resistance to ciprofloxacin and piperacillin can emerge within a patient at rates *ϵ*_1_ and *ϵ*_2_. We assume a broad range of emergence rates (0 to 3% per day).

#### Treatment and recovery

On average, we assume phenotypic resistance testing takes 3 days (*τ* = 1/3) [5], failed treatment is corrected after 3 to 5 days (*τ*_1_ ∈ [1/5, 1/3]), and 7 days of treatment is sufficient to cure infection (*τ*_2_ = 1/4). Superinfection delays recovery by 0 to 11 days (*τ*_3_ ∈ [1/4, 1/15]). Pandrug-resistance is not modelled, so we penalize dual resistance by assuming infected patients recover more slowly at a rate *ϑτ*_2_, where *ϑ* is a hazard ratio that varies broadly between 0 and 1.

#### Death, discharge and admission

Uninfected patients die at rate *ν* ∈ [0.005, 0.05] per day and are discharged at rate *μ* ∈ [0.025, 0.5] per day [52]. Infected patients recieving adequate treatment die at rate *κ*_*ν*_*ν*, and are discharged at rate *κ*_*μ*_*μ*. In general, nosocomial infections are associated with higher death rates and longer durations of stay, but the association varies among infection types (*κ*_*ν*_ ∈ [1, 2.3], *κ*_*μ*_ ∈ [0.49, 1]) [39, 40, 53]. The impact of inadequate empiric therapy on the probability of dying (*δ*) within ten days varies between 4 and 40% [41, 42]. Given *δ* and other rates we calculate the hazard ratio of death for inadequately treated patients *κ*_*δ*_ (S1 Appendix).

60% of admitted patients have prior exposure to antimicrobials (*a* = 0.6), and 0 to 10% of these are colonized with *P. aeruginosa* (*m* ∈ [0, 0.1]) [13]. Up to 70% of incoming *P. aeruginosa* strains are resistant to ciprofloxacin (*r*_1_ ∈ [0, 0.7]), and fewer are resistant to piperacillin-tazobactam (*r*_2_/*r*_1_ ∈ [0, 1]), while no MDR strains are admitted for simplicity.

The number of patients in the ICU denoted by *N* remains constant over time because that is generally the case in our ICU. To maintain a constant population we model the admission rate as a time-dependent variable *λ*(*t*) which is equal to the sum of death and discharge rates at time *t*.

### Outcomes and analysis

Nearly all relevant studies measure the impact of de-escalation on all-cause mortality [3], so we report probability of death (pDeath), measured as the percentage of patients who die in the ICU over a 100 day period. However, effects of resistance and stewardship interventions on mortality can be small and difficult to detect [3, 5, 6, 54, 55]. Infection prevalence (pInfected) and the percentage of treatment that is ineffective (pMismatch) may be more sensitive measures of the effect of de-escalation on patients.

We include several additional outcome measures to yield insight into the causes of changes in death, infection prevalence and treatment failure. A primary goal of de-escalation is to preserve empiric therapy, so we measure the percentage of empiric therapy that covers the initial infection (pEmpiric). There are concerns about emergence of superinfections and MDR bacteria and superinfections [3], so we measure the percentage of patients that have non-pseudomonal infections and *P. aeruginosa* superinfections (pSuperinf), and the prevalence of resistant to both pseudomonal drugs (pRBoth). We also measure *P. aeruginosa* colonization prevalence (pColonized).

Antimicrobial resistance is often measured as the percentage of resistant first clinical isolates [56], but we measure prevalence (pRCipro, pRPipTazo, and pRBoth) because it more accurately reflects the burden of resistance [57]. Drug use studies are common [54, 58], so we measure the use of ciprofloxacin (UseCipro), piperacillin-tazobactam (UsePipTazo), and drugs used to treat dual-resistant *P. aeruginosa* infections (UseAlt) as the percentage of patients receiving each drug. See S1 Appendix for mathematical definitions of outcomes.

To assess sensitivity we use Jansen’s estimator of the contribution of each parameter to outcome variance [59, 60]. Bootstrapped 95% confidence bounds for the total sensitivity indices indicated that a base random sample of 5000 and a total of 95,000 parameter combinations are adequate. Sampling was done using quasi-random numbers and a radial design as recommended by Saltelli et al. [59]. Parameters are assumed to be uniformly distributed over the proposed intervals because we lack information about the distributions (Table 1). This sampling scheme gives confidence that the full range of possible outcomes has been explored, at the cost of oversampling unlikely outcomes.

Two parameter sets were randomly generated for the non-pseudomonal and ciprofloxacin scenarios. For each combination of parameters, the effect of non-pseudomonal de-escalation is the difference between outcomes of the non-pseudomonal de-escalation and continuation scenarios. The ciprofloxacin de-escalation scenario is similarly compared to the continuation scenario. Outcomes are averaged over the final 100 days of a 2000-day simulation. For each combination of parameter values we numerically confirmed that the model reached a steady state.

To understand when de-escalation increases or decreases the probability of death we also analyzed a Latin-Hypercube sample of 100,000 parameter combinations using classification and regression trees [61–64]. We require a minimum of 2% of cases at a classification tree node in order to attempt a split, and pruned according to the “1-SE” rule [61]. Again, uniform distributions are assumed, and two parameter sets were randomly generated.

In our three academic ICUs over the past 14 years *P. aeruginosa* resistance to ciprofloxacin (oRCipro) and piperacillin-tazobactam (oRPipTazo) has not exceeded 70% and 50%, respectively. *P. aeruginosa* colonization prevalence ranges from 6% to 32% [65]. Outcomes outside of these ranges are considered unrealistic, and excluded from results unless otherwise specified. 34798/100000 and 33152/100000 parameter combinations meet the calibration criteria in the de-escalation and ciprofloxacin scenarios, respectively.

## Results

**Fig 3:**
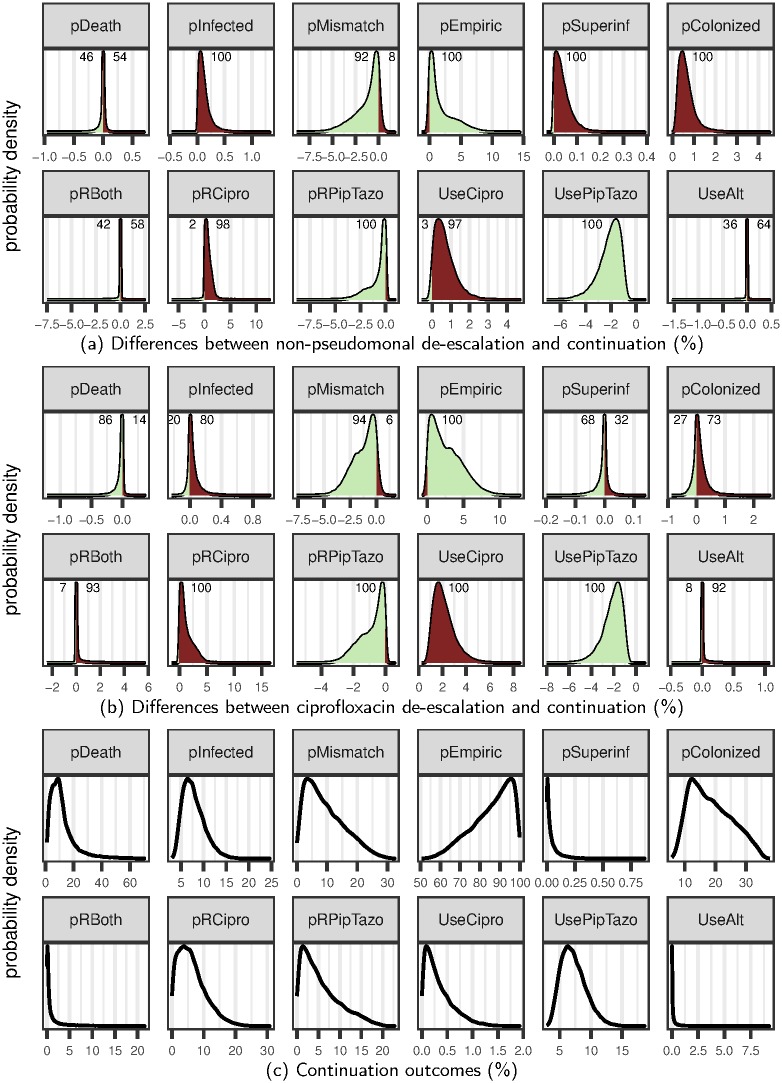
Benefits and unintended consequences of antimicrobial de-escalation. Pale green indicates a benefit of de-escalation, and dark red indicates an unintended consequence. Numbers are the percentage of cases in which de-escalation is beneficial or detrimental. Continuation outcomes (c) are shown to put observed differences in context. **pDeath** is the probability (%) of death, **pInfected** is the infection prevalence, **pMismatch** is the % of inadequate treatment, and **pEmpiric** is the % of effective empiric therapy. **pColonized** and **pSuperinf** are *P. aeruginosa* colonization and superinfection prevalence, respectively. **pRCipro/PipTazo/Both** is the prevalence of resistance, and **UseCipro/PipTazo/Alt** is drug use.

In our models de-escalation preserves the effectiveness of empiric therapy (pEmpiric in Fig 3) by decreasing the use of and resistance to piperacillin-tazobactam (UsePipTazo and pRPipTazo in Fig 3), at the cost of greater ciprofloxacin use and resistance (UseCipro and pRCipro in Fig 3). For most parameter combinations, this leads to an overall reduction in the percentage of patients that receive inadequate treatment (pMismatch in Fig 3).

In the non-pseudomonal de-escalation scenario, the benefits of de-escalation are further offset by increases in *P. aeruginosa* colonization and superinfection (pColonized and pSuperinf in Fig 3a), which in turn leads to an increase the percentage of patients suffering from an infection (pInfected in Fig 3a). Effects of de-escalation on the probability of death are small (<1%), and can be either positive or negative depending on the parameter values (pDeath in Fig 3a). In sum, de-escalation increases the probability of infection while also increasing the effectiveness of treatment (pInfected and pMismatch in Fig 3a). This tradeoff is robust to parameter uncertainty, as it occurs in nearly all cases, over a very broad range of parameter values.

In the ciprofloxacin scenario de-escalated patients remain partially protected against pseudomonal infections, so de-escalation does not consistently lead to more colonization or superinfection (pColonized and pSuperinf in Fig 3b). Instead, the benefits of de-escalation are offset by increases in dual resistance and the use of alternative pseudomonal drugs (pRBoth and UseAlt in Fig 3b). Again, these tradeoffs are robust to parameter uncertainty. For 80% of parameter combinations de-escalation still increases the percentage of infected patients (pInfected in Fig 3b). Effects of de-escalation on the probability of death remain small (<1.2%), but are more consistently beneficial (pDeath in Fig 3b).

**Fig 4:**
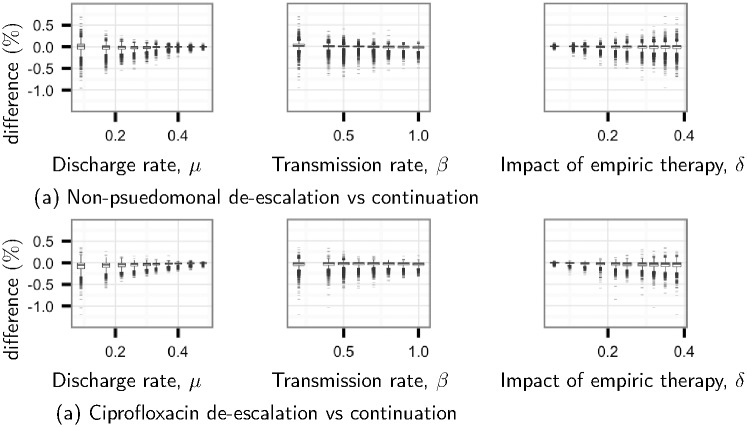
Influence of important parameters on the effect of de-escalation on the probability of death. Parameter importance was determined by sensitivity analysis (S5 Fig). Parameter values have been discretized to show variation in the distribution of outcomes—each point includes 10% of parameter combinations. Boxes show the median and interquartile range (IQR), whiskers include values within 150% of the IQR, and dashes show outliers.

**Fig 5:**
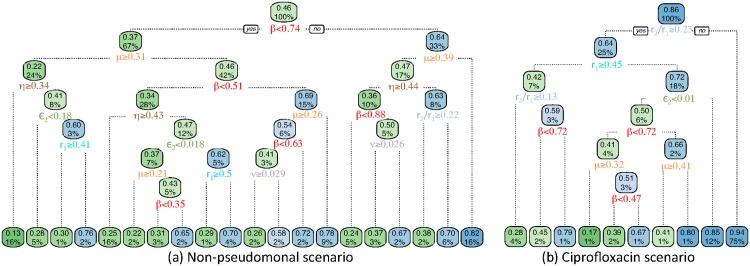
Classification trees distinguishing whether de-escalation decreases (blue) or increases (green) the probability of death. The first number at each node is the probability that de-escalation decreases the probability of death, followed by the percentage of simulations included at that node. 10-fold cross validation classification error was 25% and 11% in the (a) non-pseudomonal and (b) ciprofloxacin scenarios, respectively. Important parameters include transmission rate (*β*), discharge rate (*μ*), superinfection rate (*η*), the rate of emergence of resistance to piperacillin-tazobactam (*ϵ*_2_), incoming resistance rates (*r*_1_ and *r*_2_), and death rate (*ν*).

For many of our outcomes, de-escalation is consistently beneficial or harmful, regardless of the parameter values. The probability of death is a notable exception. De-escalation is most likely to have a substantial effect on death when discharge rate is low, transmission rate is moderate, and the impact of empiric therapy is high (Fig 4). However, other model parameters determine whether this effect is positive or negative (Fig 5).

In the non-pseudomonal scenario, determinants of whether de-escalation decreases or increases the probability of death include transmission rate (*β*), discharge rate (*μ*), superinfection rate (*η*), the rate of emergence of resistance to piperacillin-tazobactam (*ϵ*_2_), incoming resistance rates (*r*_1_ and *r*_2_), and death rate (*ν*) (Fig 5a and S6a Fig). If transmission rate (*β*) is high and discharge rate (*μ*) is low then de-escalation is likely to be beneficial (right-most branch of Fig 5a). Conversely, de-escalation is likely to be detrimental when transmission rate (*β*) is low, discharge rate (*μ*) is high, and superinfection rate (*η*) is high (left-most branch of Fig 5a). Otherwise, a complex interaction among parameters determines whether de-escalation is good or bad (Fig 5a).

In the ciprofloxacin scenario the superinfection rate (*η*) is not an important parameter and model behaviour is simpler (Fig 5b and S6b Fig). De-escalation is likely to decrease the probability of death when relatively many patients are admitted colonized by piperacillin-tazobactam resistant *P. aeruginosa* (high *r*_2_/*r*_1_) (right-most branch of S6b Fig), or when admission of resistance (*r*_1_) is low and emergence of piperacillin-tazobactam resistance (*ϵ*_2_) is high (S6b Fig).

## Discussion

We introduce novel population-level compartmental models for antimicrobial de-escalation in the ICU that distinguish between asymptomatic carriers and symptomatic patients, allow adjustment of empiric therapy, and allow the evolution of resistance to empiric and alternative drugs. A few models have allowed for adjustment of failed therapy [9–12], but none de-escalated successful empiric therapy. Our models provide insight into the potential tradeoffs associated with antimicrobial de-escalation, with implications for the design and interpretation of clinical studies.

In our models, de-escalation reduces resistance to piperacillin-tazobactam and preserves empiric therapy, as intended [1, 2, 66], at the cost of increasing resistance to ciprofloxacin. However, de-escalation to non-pseudomonal drugs also leaves patients vulnerable to *P. aeruginosa* colonization and superinfection, while de-escalation to ciprofloxacin selects for MDR. Overall, non-pseudomonal de-escalation increases the probability of adequate treatment while also increasing the probability of infection. These tradeoffs occur in nearly all the cases we investigated, across a broad range of parameter values.

Effects of de-escalation on mortality are small (<1.2%) in our models, consistent with observations [3, 5, 6]. In part this can be explained by dilution. If adequate empiric therapy can reduce 10-day mortality by up to 40%, and 17% more patients receive adequate empiric therapy, we expect a maximum impact of 6.8% which de-escalation trials have not so far been powered to detect [5, 6] (S1 Appendix). In our model, unintended consequences of de-escalation further temper the benefits of preserving empiric therapy; a 17% improvement in empiric coverage does not lead to a 6.8% reduction in the probability of death.

Increasing the probability of infection is bad for patients, while increasing the probability of adequate treatment is good for patients. To evaluate de-escalation policies it will be necessary to weigh these benefits and unintended consequences against one another. The Desirability of Outcome Ranking (DOOR) and Response Adjusted for Duration of Antibiotic Risk (RADAR) method was designed to handle competing risks, increase statistical power, and integrate harms and benefits at the patient level [58]. Integrating harms and benefits into a ranked outcome for each patient may provide a way forward in the analysis of de-escalation tradeoffs.

To date, there has been little consistency and some notable gaps in the selection of outcomes in de-escalation trials and observational studies [3, 4]. As an example, Leone et al. [5] did not measure the effectiveness of empiric therapy or MDR emergence, while Kim et al. [6] did not measure superinfections or *C. difficile* risk. No studies have adequately assessed impacts on resistance or the effectiveness of empiric therapy [3]. Proxy measures such as drug use and resistance are common in the antimicrobial stewardship literature [54, 58], but small changes in important clinical outcomes can outweigh more easily detected changes in proxy measures. We hope this study increases awareness of possible tradeoffs, and motivates more careful selection and comparison of outcomes.

In our models, the effects of de-escalation on mortality can be beneficial or detrimental. De-escalation is most likely to have a substantial effect when discharge rate is low, transmission rate is moderate, and the impact of inadequate empiric therapy is high. Superinfection rate, the rate of emergence of piperacillin-tazobactam resistance and the admission of resistant *P. aeruginosa* strains are also important. Intuitively, it makes sense that de-escalation has little effect when patient turnover is very high, when transmission is rare, when empiric therapy has little impact, or when there is little risk of resistance to empiric therapy.

Tradeoffs and parameter dependence in our results lead to a general expectation that effects of de-escalation will vary among species, contexts and patient groups. For example, most patients remain *P. aeruginosa* carriers for only 12-25 days [43], and the chance of re-infection is low. These patients may not suffer from MDR carriage after their initial infection is identified and appropriately treated. However, an important minority of immune-compromised and cystic fibrosis patients are persistent carriers of *P. aeruginosa* at high risk of reinfection, and the difficulties associated with MDR carriage are likely much higher for this group [31]. Future work might consider covering these patients with broad-spectrum antimicrobials in the high-risk ICU environment, while de-escalating lower risk patients.

Important drawbacks of ciprofloxacin de-escalation are not included in our single-species models. Ciprofloxacin is used for *P. aeruginosa* infections [5, 22–27], and it may prevent *P. aeruginosa* colonization [50]. However it can also cause complications [67] and select for resistant strains [28–30, 50]. De-escalating to a single drug would reduce prescribing heterogeneity and promote resistance among other pathogens. Ciprofloxacin and piperacillin-tazobactam are both associated with a high risk of *Clostridium difficile* infection [33–36]. De-escalation to low risk alternatives [33–36] may reduce *C. difficile* infections in patients without pseudomonal infections. Accounting for *C. difficile* risk would require a more complex model, and could tilt the balance in favour of non-pseudomonal de-escalation. The ciprofloxacin scenario clarifies model behaviour, but we do not recommend it.

The evolution of resistance is more complicated than our models. We assumed class-specific resistance mechanisms (e.g. beta-lactamases or DNA gyrase mutations) but MDR mechanisms (e.g. efflux pumps) are also common in patients with substantial antimicrobial exposure [28–32]. Including these mechanisms would reduce the impact of de-escalation on the effectiveness of empiric therapy, and thus reduce the benefits of de-escalation. Empiric therapy can include alternatives to piperacillin-tazobactam and multiple drugs [19, 20]. Stress, variation in treatment timing, drug combinations, and treatment history can alter the rate of emergence of resistance [51, 68–74]. *P. aeruginosa* persists in environmental reservoirs, and transmission dynamics are not well understood [47, 50].

It would not be difficult to build, on the basis of our compartment models, a complex individual-based model [8] that includes more drugs, more species, more infection types, more variation among patients, more transmission pathways, and more detailed evolutionary and in-host dynamics. The primary obstacle to such a model is lack of reliable parameter estimates. Even so, an individual-based model might yield some insight into *C. difficile* risk and treatment of vulnerable patients. It might also help guide the design and interpretation of studies of patient-level outcomes [58]. Our compartment models could easily be adapted for other hospital-acquired pathogens such as *Acinetobacter baumannii*. Consideration of dynamics outside the ICU will be required for species such as *E. coli* that belong to the normal human flora [75]. More complexity and realism is unlikely to alter our most important results; we expect both benefits and unintended consequences from antimicrobial de-escalation.

## Supporting information

S1 Appendix Model assumptions, equations, outcome measurements, and sample size calculation. (PDF)

S1 Fig Flow diagram of non-pseudomonal de-escalation. Compartments of patients under inadequate drug treatment are colored in orange. Discharge and death processes are not included in the diagram. See S1 Appendix for notation and equations.
(TIF)

S2 Fig Flow diagram of the continuation scenario. Compartments of patients under inadequate drug treatment are colored in orange. Discharge and death processes are not included in the diagram. See S1 Appendix for notation and equations.
(TIF)

S3 Fig Flow diagram of ciprofloxacin de-escalation. Compartments of patients under inadequate drug treatment are colored in orange. Discharge and death processes are not included in the diagram. See S1 Appendix for notation and equations.
(TIF)

S4 Fig Spearman’s rank correlations among effects of de-escalation in the (a) non-pseudomonal scenario and the (b) ciprofloxacin scenario. (TIF)

S5 Fig Total sensitivity indices [59] for the effect of de-escalation on the probability of death in the (a) non-pseudomonal and (b) ciprofloxacin scenarios. Results include parameter values that do not meet the calibration criteria.
(TIF)

S6 Fig Random Forest importance of each model parameter as a determinant of whether de-escalation decreases or increases the probability of death in the (a) non-pseudomonal and (b) ciprofloxacin scenario. 500 trees gave OOB classification error rates of 13% and 7.5% for the non-pseudomonal and ciprofloxacin scenarios, respectively.
(TIF)
