# Supplementary material for: Benefits and unintended consequences of antimicrobial de-escalation: Implications for stewardship programs
Source: PLoS One. 2017 Feb 9;12(2):e0171218. doi: 10.1371/journal.pone.0171218 (PMC5300270; doi:10.1371/journal.pone.0171218)
Supplement: S1 Appendix — (PDF) [file pone.0171218.s001.pdf]

Supplementary discussion of model notation and assumptions

Patient categorization and notation

We here provide some specific examples for the compartmental notations. $F^j_{X\to i}$ refers to patients receiving empiric therapy who were initially infected by X, colonized (but not yet infected) by strain i ($i = 2, 12$), and expected to receive definitive drug j ($j = L$ in non-pseudomonal de-escalation scenario, $j = 2$ in continuation scenario, and $j = 1$ in ciprofloxacin de-escalation scenario). $D^j_{X\to i}$ represents patients receiving definitive therapy who were infected by species X, colonized (but not yet infected) by *P.aeruginosa* strain i ($i = 0, 1, 2, 12$), and receiving definitive treatment with drug j ($j = L$ in non-pseudomonal de-escalation scenario, $j = 2$ in continuation scenario, and $j = 1$ in ciprofloxacin scenario). $S^j_{X\to i}$ represents patients superinfected by *P.aeruginosa* strain i ($i = 0, 1, 2, 12$) who are receiving drug j ($j = 1, 2, 3$).

Colonization

We ignore the persistence of sub-dominant strains. For example, consider a patient infected by a ciprofloxacin-resistant strain, D^2_1, and re-colonized by a piperacillin-tazobactam resistant strain. The first strain remains, so the patient is most likely resistant to both drugs. However, the patients do not actually carry a multi-drug resistant (MDR) strain. For simplicity, we ignore the sub-dominant strain so D^2_1 becomes D^2_2.

Infection

As an example, we illustrate our method of estimating the infection rate σ_c from literature data. We consider a group of uninfected patients, and denote their population at time t as $A(t)$. Assume these patients develop infections at the constant rate σ_c, so we have $A'(t) = -\sigma_c A(t)$ for $t \geq 0$ hence $A(t) = A(0)e^{-\sigma_c t}$. This means the percentage of patients who develop infections in t_0 days is $1 - e^{-t_0\sigma_c}$.

In the study [1] of 126 patients and 1,345 patient-days, 9 out of 20 *P.aeruginosa* colonized patients finally developed *P.aeruginosa* infection in an average of 10 days. Let $t_0 = 10$, and we have $1 - e^{-10\sigma_c} = 9/20$, hence $\sigma_c \approx 0.06\,\mathrm{day}^{-1}$. In our simulation we consider a broad range based on this estimation, letting σ_c vary between 0.05 and an upper bound of 0.14 taken from [2].

Health care and device associated infection (HCAI) incidence density ranged from 13.0 to 20.3 episodes per 1000 patient-days in high-risk adult patients in high-income countries [3]. Assuming that patients develop infection in an average of 10 days we have $1 - e^{-10\sigma_x} \in [0.13, 0.203]$. We solve the inequalities to get infection rates σ_x in the range $[0.0139, 0.0227]$.

Emergence of resistance

We do not distinguish between mutation and horizontal gene transfer [4–7], and assume the probability of developing resistance to a drug does not depend on whether a strain is resistant to the other drug . However, modification of efflux pumps and membrane permeability can alter resistance to both beta-lactams and fluoroquinolones [8–10] and exposure to low levels of ciprofloxacin increases carbapenem resistance [11]. Thus, a more realistic assumption would be that resistance to one drug increases the rate of emergence to other drugs, but does not absolutely imply resistance to the others [12].

Treatment

For most infections, treatment lasts for seven days, we assume that patients who receive adequate empiric (for an average of 3 days) and definitive therapy (for an average of 4 days) recover at a rate $\tau_2 = 1/4$ per day. For simplicity, we assume that all patients receiving effective definitive treatment recover at rate τ_2, regardless of whether their empiric treatment was adequate.

The impact of ineffective empiric therapy

In order to get a range of plausible values for the hazard ratio of death (κ_δ), we use the estimations of the impact of ineffective empiric therapy (δ) from literatures. Two recent reviews confirm that inappropriate empiric therapy increases mortality of critically ill patients [13, 14]. Among studies of *P.aeruginosa* VAP or HAP infections, the impact of inappropriate empiric therapy on the probability of death varied between 4% [15] and 40% [16].

Consider a simple scenario in which patients recover after 7 days of effective treatment, empiric therapy lasts for 3 days, and definitive therapy is always adequate. Patients receiving adequate empiric therapy recover after 7 days, and patients receiving inadequate empiric therapy recover after 10 days. Given the parameters ν (death rate without nosocomial infection) and κ_ν (hazard ratio of death with a nosocomial infection), we can calculate the probability of death in a 10-day ICU stay with effective empiric therapy (7-day effective therapy and 3-day uninfected) is $1 - e^{-7\nu\kappa_\nu - 3\nu}$, and that with ineffective empiric therapy (3-day ineffective therapy and 7-day effective therapy) is $1 - e^{-3\nu\kappa_\nu\kappa_\delta - 7\nu\kappa_\nu}$. Then δ is the difference between the above death probabilities, which is

$$\delta = e^{-7\nu\kappa_\nu - 3\nu}\left[1 - e^{3\nu(1-\kappa_\nu\kappa_\delta)}\right].$$

For each value of ν and κ_ν we calculate the range κ_δ that gives a range of δ from 4% to 40%. We use the derived range of κ_δ in the model, and interpret δ because it is a more intuitively meaningful summary of the impact of ineffective empiric therapy.

Model equations

For the convenience of presenting the equations, we group the population compartments according to different attributes. To group the population with respect to their infection and treatment status, we denote U as all uninfected patients, E as infected patients receiving empiric therapy without strain conversion, F as infected patients receiving empiric therapy with strain conversion, D as infected patients receiving definitive therapy, and S as patients superinfected by *P. aeruginosa*.

To group patients according to their colonization status, we denote R_0, R_1, R_2, R_{12} as patients colonized or infected with strains that are susceptible to all drugs, resistant only to ciprofloxaxin, resistant only to piperacillin-tazobactam, and resistant to both drugs, respectively.

To distinguish patients with respect to their treatment effectiveness, we denote P_{eff} as patients under effective treatments, and P_{ineff} as patients under ineffective treatments.

The compartment compositions for most of the above groups are different in each scenario, except for that of U and E which are status unrelated to drug use strategies, so we list them here for all three scenarios:

$$\begin{aligned} U &:= U_X^N + U_X^A + U_0^A + U_1^A + U_2^A + U_{12}^A \\ E &:= E_X^2 + E_0^2 + E_1^2 + E_2^2 + E_{12}^2 \end{aligned}$$

Non-pseudomonal de-escalation scenario

We first list the population groups for this scenario:

$$\begin{aligned}
F &= F_{X\to2}^L + F_{X\to12}^L + F_2^1 + F_{12}^1 + F_2^2 + F_{12}^2 \\
D &= D_X^L + D_0^L + D_1^L + D_{X\to2}^L + D_{X\to12}^L + D_0^1 + D_1^1 + D_{12}^1 + D_2^1 + D_1^2 + D_2^2 + D_{12}^2 + D_{12}^3 \\
S &= S_{X\to1}^2 + S_{X\to0}^1 + S_{X\to2}^1 + S_{X\to12}^3 \\
R_0 &= U_0^A + E_0^2 + D_0^L + D_0^1 + S_{X\to0}^1 \\
R_1 &= U_1^A + E_1^2 + D_1^L + D_1^1 + D_1^2 + S_{X\to1}^2 \\
R_2 &= U_2^A + E_2^2 + F_{X\to2}^L + F_2^1 + F_2^2 + D_{X\to2}^L + D_2^1 + D_2^2 + S_{X\to2}^1 \\
R_{12} &= U_{12}^A + E_{12}^2 + F_{X\to12}^L + F_{12}^1 + F_{12}^2 + D_{X\to12}^L + D_{12}^1 + D_{12}^2 + D_{12}^3 + S_{X\to12}^3 \\
P_{\text{eff}} &= E_X^2 + E_0^2 + E_1^2 + F_{X\to2}^L + F_{X\to12}^L + D_X^L + D_0^L + D_1^L + D_{X\to2}^L + D_{X\to12}^L + D_0^1 + D_2^1 + D_1^2 + D_{12}^3 \\
P_{\text{ineff}} &= E_2^2 + E_{12}^2 + F_{12}^1 + F_2^1 + F_2^2 + F_{12}^2 + D_1^1 + D_{12}^1 + D_2^2 + D_{12}^2 + S_{X\to1}^2 + S_{X\to0}^1 + S_{X\to2}^1 + S_{X\to12}^3
\end{aligned}$$

The non-pseudomonal de-escalation scenario model (S1 Fig) is governed by the following system of 35 ordinary

differential equations:

$$\lambda(t) = (\mu+\nu)U(t) + (\kappa_\mu\mu + \kappa_\nu\nu)P_{\text{eff}}(t) + (\kappa_\mu\mu + \kappa_\delta\kappa_\nu\nu)P_{\text{ineff}}(t) \tag{A.1}$$

$$\dot{U}_X^N(t) = (1-a)\lambda(t) - (\mu+\nu+\sigma_x)U_X^N(t) \tag{A.2}$$

$$\dot{U}_X^A(t) = (1-m)a\lambda(t) + \tau_2 D_X^L(t) - \beta\Big(R_0(t)+R_1(t)+R_2(t)+R_{12}(t)\Big)U_X^A(t)/N - (\mu+\nu+\sigma_x)U_X^A(t) \tag{A.3}$$

$$\dot{U}_0^A(t) = (1-r_1-r_2)ma\lambda(t) + \beta R_0(t)U_X^A(t)/N + \tau_2 D_0^1(t) + (1-\eta)\tau_2 D_0^L(t) + \tau_3 S_{X\to0}^1(t) - (\mu+\nu+\sigma_c)U_0^A(t) \tag{A.4}$$

$$\dot{U}_1^A(t) = r_1 ma\lambda(t) + \beta R_1(t)U_X^A(t)/N + \tau_2 D_1^2(t) + (1-\eta)\tau_2 D_1^L(t) + \tau_3 S_{X\to1}^2(t) - (\mu+\nu+\sigma_c)U_1^A(t) \tag{A.5}$$

$$\dot{U}_2^A(t) = r_2 ma\lambda(t) + \beta R_2(t)U_X^A(t)/N + \tau_2 D_2^1(t) + (1-\eta)\tau_2 D_{X\to2}^L(t) + \tau_3 S_{X\to2}^1(t) - (\mu+\nu+\sigma_c)U_2^A(t) \tag{A.6}$$

$$\dot{U}_{12}^A(t) = \vartheta\tau_2 D_{12}^3(t) + \beta R_{12}(t)U_X^A(t)/N + (1-\eta)\tau_2 D_{X\to12}^L(t) + \tau_3 S_{X\to12}^3(t) - (\mu+\nu+\sigma_c)U_{12}^A(t) \tag{A.7}$$

$$\dot{E}_X^2(t) = \sigma_x\Big(U_X^N(t)+U_X^A(t)\Big) - \beta\Big(R_2(t)+R_{12}(t)\Big)E_X^2(t)/N - (\tau+\kappa_\mu\mu+\kappa_\nu\nu)E_X^2(t) \tag{A.8}$$

$$\dot{E}_0^2(t) = \sigma_c U_0^A(t) - \beta\Big(R_2(t)+R_{12}(t)\Big)E_0^2(t)/N - (\tau+\kappa_\mu\mu+\kappa_\nu\nu+\varepsilon_2)E_0^2(t) \tag{A.9}$$

$$\dot{E}_1^2(t) = \sigma_c U_1^A(t) - \beta\Big(R_2(t)+R_{12}(t)\Big)E_1^2(t)/N - (\tau+\kappa_\mu\mu+\kappa_\nu\nu+\varepsilon_2)E_1^2(t) \tag{A.10}$$

$$\dot{E}_2^2(t) = \sigma_c U_2^A(t) - (\tau+\kappa_\mu\mu+\kappa_\delta\kappa_\nu\nu)E_2^2(t) \tag{A.11}$$

$$\dot{E}_{12}^2(t) = \sigma_c U_{12}^A(t) - (\tau+\kappa_\mu\mu+\kappa_\delta\kappa_\nu\nu)E_{12}^2(t) \tag{A.12}$$

$$\dot{F}_{X\to2}^L(t) = \beta R_2(t)E_X^2(t)/N - (\tau+\kappa_\mu\mu+\kappa_\nu\nu)F_{X\to2}^L(t) \tag{A.13}$$

$$\dot{F}_{X\to12}^L(t) = \beta R_{12}(t)E_X^2(t)/N - (\tau+\kappa_\mu\mu+\kappa_\nu\nu)F_{X\to12}^L(t) \tag{A.14}$$

$$\dot{F}_2^1(t) = \beta R_2(t)E_0^2(t)/N + \varepsilon_2 E_0^2(t) - (\tau+\kappa_\mu\mu+\kappa_\delta\kappa_\nu\nu)F_2^1(t) \tag{A.15}$$

$$\dot{F}_{12}^1(t) = \beta R_{12}(t)E_0^2(t)/N - (\tau+\kappa_\mu\mu+\kappa_\delta\kappa_\nu\nu)F_{12}^1(t) \tag{A.16}$$

$$\dot{F}_2^2(t) = \beta R_2(t)E_1^2(t)/N - (\tau+\kappa_\mu\mu+\kappa_\delta\kappa_\nu\nu)F_2^2(t) \tag{A.17}$$

$$\dot{F}_{12}^2(t) = \beta R_{12}(t)E_1^2(t)/N + \varepsilon_2 E_1^2(t) - (\tau+\kappa_\mu\mu+\kappa_\delta\kappa_\nu\nu)F_{12}^2(t) \tag{A.18}$$

$$\dot{D}_X^L(t) = \tau E_X^2(t) - \beta\Big(R_0(t)+R_1(t)+R_2(t)+R_{12}(t)\Big)D_X^L(t)/N - (\kappa_\mu\mu+\kappa_\nu\nu+\tau_2)D_X^L(t) \tag{A.19}$$

$$\dot{D}_0^L(t) = \beta R_0(t)D_X^L(t)/N - (\kappa_\mu\mu+\kappa_\nu\nu+\eta\tau_1+(1-\eta)\tau_2)D_0^L(t) \tag{A.20}$$

$$\dot{D}_1^L(t) = \beta R_1(t)D_X^L(t)/N - (\kappa_\mu\mu+\kappa_\nu\nu+\eta\tau_1+(1-\eta)\tau_2)D_1^L(t) \tag{A.21}$$

$$\dot{D}_{X\to2}^L(t) = \beta R_2(t)D_X^L(t)/N + \tau F_{X\to2}^L(t) - (\kappa_\mu\mu+\kappa_\nu\nu+\eta\tau_1+(1-\eta)\tau_2)D_{X\to2}^L(t) \tag{A.22}$$

$$\dot{D}_{X\to12}^L(t) = \beta R_{12}(t)D_X^L(t)/N + \tau F_{X\to12}^L(t) - (\kappa_\mu\mu+\kappa_\nu\nu+\eta\tau_1+(1-\eta)\tau_2)D_{X\to12}^L(t) \tag{A.23}$$

$$\dot{D}_0^1(t) = \tau E_0^2(t) - \beta\Big(R_1(t)+R_{12}(t)\Big)D_0^1(t)/N - (\kappa_\mu\mu+\kappa_\nu\nu+\tau_2+\varepsilon_1)D_0^1(t) \tag{A.24}$$

$$\dot{D}_1^1(t) = \beta R_1(t)\Big(D_0^1(t)+D_2^1(t)\Big)/N + \varepsilon_1 D_0^1(t) - (\kappa_\mu\mu+\kappa_\delta\kappa_\nu\nu+\tau_1)D_1^1(t) \tag{A.25}$$

$$\dot{D}_2^1(t) = \tau E_2^2(t) + \tau F_2^1(t) + \tau_1 D_2^2(t) - \beta\Big(R_1(t)+R_{12}(t)\Big)D_2^1(t)/N - (\kappa_\mu\mu+\kappa_\nu\nu+\tau_2+\varepsilon_1)D_2^1(t) \tag{A.26}$$

$$\dot{D}_{12}^1(t) = \tau F_{12}^1(t) + \beta R_{12}(t)\Big(D_0^1(t)+D_2^1(t)\Big)/N + \varepsilon_1 D_2^1(t) - (\kappa_\mu\mu+\kappa_\delta\kappa_\nu\nu+\tau_1)D_{12}^1(t) \tag{A.27}$$

$$\dot{D}_1^2(t) = \tau E_1^2(t) + \tau_1 D_1^1(t) - \beta\Big(R_2(t)+R_{12}(t)\Big)D_1^2(t)/N - (\kappa_\mu\mu+\kappa_\nu\nu+\varepsilon_2+\tau_2)D_1^2(t) \tag{A.28}$$

$$\dot{D}_2^2(t) = \tau F_2^2(t) + \beta R_2(t)D_1^2(t)/N - (\kappa_\mu\mu+\kappa_\delta\kappa_\nu\nu+\tau_1)D_2^2(t) \tag{A.29}$$

$$\dot{D}_{12}^2(t) = \tau F_{12}^2(t) + \beta R_{12}(t)D_1^2(t)/N + \varepsilon_2 D_1^2(t) - (\kappa_\mu\mu+\kappa_\delta\kappa_\nu\nu+\tau_1)D_{12}^2(t) \tag{A.30}$$

$$\dot{D}_{12}^3(t) = \tau E_{12}^2(t) + \tau_1\Big(D_{12}^1(t)+D_{12}^2(t)\Big) - (\kappa_\mu\mu+\kappa_\nu\nu+\vartheta\tau_2)D_{12}^3(t) \tag{A.31}$$

$$\dot{S}_{X\to1}^2(t) = \eta\tau_1 D_1^L(t) - (\tau_3+\kappa_\mu\mu+\kappa_\delta\kappa_\nu\nu)S_{X\to1}^2(t) \tag{A.32}$$

$$\dot{S}_{X\to0}^1(t) = \eta\tau_1 D_0^L(t) - (\tau_3+\kappa_\mu\mu+\kappa_\delta\kappa_\nu\nu)S_{X\to0}^1(t) \tag{A.33}$$

$$\dot{S}_{X\to2}^1(t) = \eta\tau_1 D_{X\to2}^L(t) - (\tau_3+\kappa_\mu\mu+\kappa_\delta\kappa_\nu\nu)S_{X\to2}^1(t) \tag{A.34}$$

$$\dot{S}_{X\to12}^3(t) = \eta\tau_1 D_{X\to12}^L(t) - (\tau_3+\kappa_\mu\mu+\kappa_\delta\kappa_\nu\nu)S_{X\to12}^3(t) \tag{A.35}$$

Continuation scenario

The population categorization is listed as follows:

$$
\begin{aligned}
&F = F_2^2 + F_{12}^2 + F_{X\to 2}^2 + F_{X\to 12}^2 \\
&D = D_X^2 + D_{X\to 2}^2 + D_{X\to 12}^2 + D_2^2 + D_0^2 + D_{12}^2 + D_1^2 + D_2^1 + D_1^1 + D_{12}^1 + D_{12}^3 \\
&S = S_{X\to 2}^1 + S_{X\to 12}^3 \\
&R_0 = U_0^A + E_0^2 + D_0^2 \\
&R_1 = U_1^A + E_1^2 + D_1^2 + D_1^1 \\
&R_2 = U_2^A + E_2^2 + F_2^2 + F_{X\to 2}^2 + D_2^2 + D_2^1 + D_{X\to 2}^2 + S_{X\to 2}^1 \\
&R_{12} = U_{12}^A + E_{12}^2 + F_{12}^2 + F_{X\to 12}^2 + D_{12}^1 + D_{12}^2 + D_{12}^3 + D_{X\to 12}^2 + S_{X\to 12}^3 \\
&P_{\text{eff}} = E_X^2 + E_0^2 + E_1^2 + F_{X\to 2}^2 + F_{X\to 12}^2 + D_X^2 + D_{X\to 2}^2 + D_{X\to 12}^2 + D_0^2 + D_1^2 + D_2^1 + D_{12}^3 \\
&P_{\text{ineff}} = E_2^2 + E_{12}^2 + F_2^2 + F_{12}^2 + D_1^1 + D_2^2 + D_{12}^1 + D_{12}^2 + S_{X\to 2}^1 + S_{X\to 12}^3
\end{aligned}
$$

The system of ordinary differential equations for the continuation scenario (S2 Fig) is governed by the following equations:

$$\lambda(t) = (\mu+\nu)U(t) + (\kappa_\mu\mu + \kappa_\nu\nu)P_{\text{eff}}(t) + (\kappa_\mu\mu + \kappa_\delta\kappa_\nu\nu)P_{\text{ineff}}(t) \tag{A.36}$$

$$\dot{U}_X^N(t) = (1-a)\lambda(t) - (\mu+\nu+\sigma_x)U_X^N(t) \tag{A.37}$$

$$\dot{U}_X^A(t) = (1-m)a\lambda(t) + \tau_2 D_X^2(t) - \beta\Big(R_0(t) + R_1(t) + R_2(t) + R_{12}(t)\Big)U_X^A(t)/N - (\mu+\nu+\sigma_x)U_X^A(t) \tag{A.38}$$

$$\dot{U}_0^A(t) = (1-r_1-r_2)ma\lambda(t) + \beta R_0(t)U_X^A(t)/N + \tau_2 D_0^2(t) - (\mu+\nu+\sigma_c)U_0^A(t) \tag{A.39}$$

$$\dot{U}_1^A(t) = r_1 ma\lambda(t) + \beta R_1(t)U_X^A(t)/N + \tau_2 D_1^2(t) - (\mu+\nu+\sigma_c)U_1^A(t) \tag{A.40}$$

$$\dot{U}_2^A(t) = r_2 ma\lambda(t) + \beta R_2(t)U_X^A(t)/N + \tau_2\left(D_2^1(t) + (1-\eta)D_{X\to 2}^2(t)\right) + \tau_3 S_{X\to 2}^1(t) - (\mu+\nu+\sigma_c)U_2^A(t) \tag{A.41}$$

$$\dot{U}_{12}^A(t) = \vartheta\tau_2 D_{12}^3(t) + \tau_2(1-\eta)D_{X\to 12}^2(t) + \beta R_{12}(t)U_X^A(t)/N + \tau_3 S_{X\to 12}^3(t) - (\mu+\nu+\sigma_c)U_{12}^A(t) \tag{A.42}$$

$$\dot{E}_X^2(t) = \sigma_x\Big(U_X^N(t) + U_X^A(t)\Big) - \beta\Big(R_2(t) + R_{12}(t)\Big)E_X^2(t)/N - (\tau + \kappa_\mu\mu + \kappa_\nu\nu)E_X^2(t) \tag{A.43}$$

$$\dot{E}_0^2(t) = \sigma_c U_0^A(t) - \beta\Big(R_2(t) + R_{12}(t)\Big)E_0^2(t)/N - (\tau + \kappa_\mu\mu + \kappa_\nu\nu + \varepsilon_2)E_0^2(t) \tag{A.44}$$

$$\dot{E}_1^2(t) = \sigma_c U_1^A(t) - \beta\Big(R_2(t) + R_{12}(t)\Big)E_1^2(t)/N - (\tau + \kappa_\mu\mu + \kappa_\nu\nu + \varepsilon_2)E_1^2(t) \tag{A.45}$$

$$\dot{E}_2^2(t) = \sigma_c U_2^A(t) - (\tau + \kappa_\mu\mu + \kappa_\delta\kappa_\nu\nu)E_2^2(t) \tag{A.46}$$

$$\dot{E}_{12}^2(t) = \sigma_c U_{12}^A(t) - (\tau + \kappa_\mu\mu + \kappa_\delta\kappa_\nu\nu)E_{12}^2(t) \tag{A.47}$$

$$\dot{F}_{X\to 2}^2(t) = \beta R_2(t)E_X^2(t)/N - (\tau + \kappa_\mu\mu + \kappa_\nu\nu)F_{X\to 2}^2(t) \tag{A.48}$$

$$\dot{F}_{X\to 12}^2(t) = \beta R_{12}(t)E_X^2(t)/N - (\tau + \kappa_\mu\mu + \kappa_\nu\nu)F_{X\to 12}^2(t) \tag{A.49}$$

$$\dot{F}_2^2(t) = \beta R_2(t)\left(E_0^2(t) + E_1^2(t)\right)/N + \varepsilon_2 E_0^2(t) - (\tau + \kappa_\mu\mu + \kappa_\delta\kappa_\nu\nu)F_2^2(t) \tag{A.50}$$

$$\dot{F}_{12}^2(t) = \beta R_{12}(t)\left(E_0^2(t) + E_1^2(t)\right)/N + \varepsilon_2 E_1^2(t) - (\tau + \kappa_\mu\mu + \kappa_\delta\kappa_\nu\nu)F_{12}^2(t) \tag{A.51}$$

$$\dot{D}_X^2(t) = \tau E_X^2(t) - \beta\left(R_2(t) + R_{12}(t)\right)D_X^2(t)/N - (\tau_2 + \kappa_\mu\mu + \kappa_\nu\nu)D_X^2(t) \tag{A.52}$$

$$\dot{D}_{X\to 2}^2(t) = \tau F_{X\to 2}^2(t) + \beta R_2(t)D_X^2(t)/N - (\kappa_\mu\mu + \kappa_\nu\nu + \eta\tau_1 + (1-\eta)\tau_2)D_{X\to 2}^2(t) \tag{A.53}$$

$$\dot{D}^2_{X\to12}(t) = \tau F^2_{X\to12}(t) + \beta R_{12}(t) D^2_X(t)/N - \left(\kappa_\mu\mu + \kappa_\nu\nu + \eta\tau_1 + (1-\eta)\tau_2\right) D^2_{X\to12}(t) \tag{A.54}$$

$$\dot{D}^2_2(t) = \tau F^2_2(t) + \beta R_2(t)\left(D^2_0(t) + D^2_1(t)\right)/N + \varepsilon_2 D^2_0(t) - \left(\tau_1 + \kappa_\mu\mu + \kappa_\delta\kappa_\nu\nu\right) D^2_2(t) \tag{A.55}$$

$$\dot{D}^2_0(t) = \tau E^2_0(t) - \beta\left(R_2(t) + R_{12}(t)\right) D^2_0(t)/N - \left(\varepsilon_2 + \tau_2 + \kappa_\mu\mu + \kappa_\nu\nu\right) D^2_0(t) \tag{A.56}$$

$$\dot{D}^2_{12}(t) = \tau F^2_{12}(t) + \beta R_{12}(t)\left(D^2_0(t) + D^2_1(t)\right)/N + \varepsilon_2 D^2_1(t) - \left(\tau_1 + \kappa_\mu\mu + \kappa_\delta\kappa_\nu\nu\right) D^2_{12}(t) \tag{A.57}$$

$$\dot{D}^2_1(t) = \tau E^2_1(t) + \tau_1 D^1_1(t) - \beta\left(R_2(t) + R_{12}(t)\right) D^2_1(t)/N - \left(\varepsilon_2 + \tau_2 + \kappa_\mu\mu + \kappa_\nu\nu\right) D^2_1(t) \tag{A.58}$$

$$\dot{D}^1_2(t) = \tau E^2_2(t) + \tau_1 D^2_2(t) - \beta\left(R_1(t) + R_{12}(t)\right) D^1_2(t)/N - \left(\varepsilon_1 + \tau_2 + \kappa_\mu\mu + \kappa_\nu\nu\right) D^1_2(t) \tag{A.59}$$

$$\dot{D}^1_1(t) = \beta R_1(t) D^1_2(t)/N - \left(\tau_1 + \kappa_\mu\mu + \kappa_\delta\kappa_\nu\nu\right) D^1_1(t) \tag{A.60}$$

$$\dot{D}^1_{12}(t) = \beta R_{12}(t) D^1_2(t)/N + \varepsilon_1 D^1_2(t) - \left(\tau_1 + \kappa_\mu\mu + \kappa_\delta\kappa_\nu\nu\right) D^1_{12}(t) \tag{A.61}$$

$$\dot{D}^3_{12}(t) = \tau E^2_{12}(t) + \tau_1\left(D^1_{12}(t) + D^2_{12}(t)\right) - \left(\vartheta\tau_2 + \kappa_\mu\mu + \kappa_\nu\nu\right) D^3_{12}(t) \tag{A.62}$$

$$\dot{S}^1_{X\to2}(t) = \eta\tau_1 D^2_{X\to2}(t) - (\tau_3 + \kappa_\mu\mu + \kappa_\delta\kappa_\nu\nu) S^1_{X\to2}(t) \tag{A.63}$$

$$\dot{S}^3_{X\to12}(t) = \eta\tau_1 D^2_{X\to12}(t) - (\tau_3 + \kappa_\mu\mu + \kappa_\delta\kappa_\nu\nu) S^3_{X\to12}(t) \tag{A.64}$$

Ciprofloxacin de-escalation scenario

The ciprofloxacin de-escalation scenario (S3 Fig) is a special case of the non-pseudomonal de-escalation. Replace D^L_X by D^1_X, D^L_1 by $D^1_{X\to1}$, $D^L_{X\to2}$ by $D^1_{X\to2}$, and $D^L_{X\to12}$ by $D^1_{X\to12}$. Set $D^L_0 = S^1_{X\to0} = S^1_{X\to2} = 0$.

Outcome measurement

Simulations suggest a single stable equilibrium. Due to the high dimensionality and non-linearity of the system, we cannot explicitly solve for that equilibrium. Instead we measure outcomes by computing 100-day average after a sufficiently long burn-in period. For each parameter set, we begin with an ICU that does not contain any resistance, simulate 2000 days, and take the average of each compartment over the final 100 days. For each combination of parameter values we numerically confirmed that the model reached a steady state Outcomes are defined in table A.1.

Sample Size Calculation

For a random variable with standardised normal distribution, $Z_{1-\alpha/2}$ is denoted as the $1-\alpha/2$-th percentile value - the threshold value for which the probability of the random variable having a value that exceeds $Z_{1-\alpha/2}$ is $\alpha/2$. Based on an initial ICU mortality rate of 20% ($p_1 = 0.2$) in Ontario teaching hospitals [17], for a 95% confidence ($\alpha = 0.05$), and a 90% of power ($\beta = 0.1$), the sample size (n) needed to detect a 6.8% change in death rate ($p_2 = 0.132$) via a two-arm clinical trial is computed as follows [18, 19]:

$$\begin{aligned} n =& \frac{p_1(1-p_1) + p_2(1-p_2)}{(p_1-p_2)^2}\left(Z_{1-\alpha/2} + Z_{1-\beta}\right)^2 \\ \approx& \; 636 \text{ per arm} \end{aligned}$$

Sample size estimation implies to detect this difference 1272 participants would be needed in a two-arm clinical trial. This value would be increased in a cluster randomized control trial [18].

References

1. Boyer A, Doussau A, Thiebault R, Venier AG, Tran V, Boulestreau H, et al. Pseudomonas aeruginosa acquisition on an intensive care unit: relationship between antibiotic selective pressure and patients' environment. Crit Care. 2011;15(1):R55.

2. Hurford A, Morris AM, Fisman DN, Wu JH. Linking antimicrobial prescribing to antimicrobial resistance in the ICU: Before and after an antimicrobial stewardship program. Epidemics. 2012;4(4):203–210.

Table A.1. Mathematical definitions of outcome measurements

Abbreviation	Measurement (100-day average in %)
pDeath	# of deaths in 100 days/# of admissions in 100 days
	$^{*}\ \odot\ ^{\dagger}$ $100 \times (\kappa_\delta \kappa_\nu \nu P_{\text{ineff}} + \kappa_\nu \nu P_{\text{eff}} + \nu U)/\lambda$
pInfected	# of infected patients/total # of patients
	$^{*}\odot^{\dagger}$ $100 \times (E + F + D + S)/N$
pColonized	# of patients colonized with *P. aeruginosa*/total # of patients
	* $100 \times \big(1 - (U_X^N + U_X^A + E_X^2 + D_X^L)/N\big)$
	$\odot$ $100 \times \big(1 - (U_X^N + U_X^A + E_X^2 + D_X^2)/N\big)$
	† $100 \times \big(1 - (U_X^N + U_X^A + E_X^2 + D_X^1)/N\big)$
pRCipro	patients colonized or infected with strains resistant to cipro/total # of patients
	$^{*}\ \odot\ ^{\dagger}$ $100 \times (R_1 + R_{12})/N$
pRPipTazo	patients colonized or infected with strains resistant to pip-tazo/total # of patients
	$^{*}\ \odot\ ^{\dagger}$ $100 \times R_2 + R_{12}/N$
pRBoth	patients colonized or infected with strains resistant to cipro and pip-tazo/total # of patients
	$^{*}\ \odot\ ^{\dagger}$ $100 \times R_{12}/N$
UseCipro	# of patients taking cipro/total # of patients
	* $100 \times (D_0^1 + D_1^1 + D_2^1 + D_{12}^1 + S_{X\to 0}^1 + S_{X\to 2}^1)/N$
	$\odot$ $100 \times (D_1^1 + D_2^1 + D_{12}^1 + S_{X\to 2}^1)/N$
	† $100 \times (D_0^1 + D_1^1 + D_2^1 + D_{12}^1 + D_X^1 + D_{X\to 1}^1 + D_{X\to 2}^1 + D_{X\to 12}^1)/N$
UsePipTazo	# of patients taking pip-tazo/total # of patients
	* $100 \times (E + F + D_1^2 + D_2^2 + D_{12}^2 + S_{X\to 1}^2)/N$
	$\odot$ $100 \times (E + F + D_{X\to 2}^2 + D_{X\to 12}^2 + D_0^2 + D_1^2 + D_2^2 + D_{12}^2)/N$
	† $100 \times (E + F + D_1^2 + D_2^2 + D_{12}^2 + S_{X\to 1}^2)/N$
UseAlt	# of patients taking alternative drug 3/total # of patients
	$^{*}\ \odot\ ^{\dagger}$ $100 \times (D_{12}^3 + S_{X\to 12}^3)/N$
pEmpiric	# of patients empirically treated with a drug that covers the initial infecting pathogen/# of patients in empiric therapy
	$^{*}\ \odot\ ^{\dagger}$ $100 \times \big(1 - (E_2^2 + E_{12}^2)/(E + F)\big)$
pSuperinf	# of superinfected patients/total # of patients
	$^{*}\ \odot\ ^{\dagger}$ $100 \times S/N$
pMisMatch	# of patients treated with an ineffective drug/# of patients under antibiotic treatment
	$^{*}\ \dagger$ $100 \times \big(E_2^2 + E_{12}^2 + F_2^1 + F_{12}^1 + F_2^2 + F_{12}^2 + D_1^1 + D_2^2 + D_{12}^1 + D_{12}^2\big)/\big(E + F + D + S\big)$
	$\odot$ $100 \times \big(E_2^2 + E_{12}^2 + F_2^2 + F_{12}^2 + D_1^1 + D_2^2 + D_{12}^1 + D_{12}^2\big)/\big(E + F + D + S\big)$

* - non-pseudomonal de-escalation
$^{\odot}$ - continuation
† - ciprofloxacin de-escalation
Average values for each compartment are taken over the final 100 days of a 2000 day simulation.

3. WHO: World Health Organization. Report on the burden of endemic health care-associated infection worldwide: A systematic review of the literature; 2011. Available from: `http://apps.who.int/iris/bitstream/10665/80135/1/9789241501507_eng.pdf?ua=1`.

4. Gehring R, Schumm P, Youssef M, Scoglio C. A network-based approach for resistance transmission in bacterial populations. J Theor Biol. 2010;262(1):97–106.

5. Geli P, Laxminarayan R, Dunne M, Smith DL. "One-Size-Fits-All"? Optimizing treatment duration for bacterial infections. PLoS One. 2012;7(1).

6. MacLean RC, Hall AR, Perron GG, Buckling A. The population genetics of antibiotic resistance: integrating molecular mechanisms and treatment contexts. Nat Rev Genet. 2010;11(6):405–414.

7. Poole K. Pseudomonas aeruginosa: resistance to the max. Front Microbiol. 2011;2. doi:10.3389/fmicb.2011.00065.

8. Poole K. Stress responses as determinants of antimicrobial resistance in Pseudomonas aeruginosa: multidrug efflux and more. Can J Microbiol. 2014;60(12):783–791.

9. Breidenstein EB, de la Fuente-Nunez C, Hancock RE. Pseudomonas aeruginosa: all roads lead to resistance. Trends Microbiol. 2011;19(8):419–426.

10. Mesaros N, Nordmann P, Plesiat P, Roussel-Delvallez M, Van Eldere J, Glupczynski Y, et al. Pseudomonas aeruginosa: resistance and therapeutic options at the turn of the new millennium. Clin Microbiol Infec. 2007;13(6):560–578.

11. Morita Y, Tomida J, Kawamura Y. Responses of Pseudomonas aeruginosa to antimicrobials. Front Microbiol. 2014;4. doi:10.3389/fmicb.2013.00422.

12. Wong PHP, von Krosigk M, Roscoe DL, Lau TTY, Yousefi M, Bowie WR. Antimicrobial co-resistance patterns of gram-negative bacilli isolated from bloodstream infections: a longitudinal epidemiological study from 2002-2011. BMC Infect Dis. 2014;14:393. doi:10.1186/1471-2334-14-393.

13. Marquet K, Liesenborgs A, Bergs J, Vleugels A, Claes N. Incidence and outcome of inappropriate in-hospital empiric antibiotics for severe infection: a systematic review and meta-analysis. Crit Care. 2015;19(1):63.

14. Paul M, Shani V, Muchtar E, Kariv G, Robenshtok E, Leibovici L. Systematic Review and Meta-Analysis of the Efficacy of Appropriate Empiric Antibiotic Therapy for Sepsis. Antimicrob Agents Chemother. 2010;54(11):4851–4863.

15. Heyland DK, Cook DJ, Griffith L, Keenan SP, Brun-Buisson C. The attributable morbidity and mortality of ventilator-associated pneumonia in the critically ill patient. The Canadian Critical Trials Group. Am J Respir Crit Care Med. 1999;159(4 Pt 1):1249–1256. doi:10.1164/ajrccm.159.4.9807050.

16. Tumbarello M, De Pascale G, Trecarichi EM, Spanu T, Antonicelli F, Maviglia R, et al. Clinical outcomes of Pseudomonas aeruginosa pneumonia in intensive care unit patients. Intensive Care Med. 2013;39(4):682–692. doi:10.1007/s00134-013-2828-9.

17. DiDiodato G. Comparison of Ontario's Intensive Care Units' Reported Mortality Rates From 2009–2012 Using Data From the Critical Care Information System (CCIS); 2012. Available from: http://www.criticalcarecanada.com/abstracts/2012/comparison_of_ontarios_intensive_care_units_reported_mortality_rates_from_2009-2012_using_data_from_the_critical_care_information_system.php.

18. Rutterford C, Copas A, Eldridge S. Methods for sample size determination in cluster randomized trials. Int J Epidemiol. 2015;44(3):1051–1067. doi:10.1093/ije/dyv113.

19. Dean AG, Sullivan KM, Soe MM. OpenEpi: Open source epidemiologic statistics for public health, version; 2014.
