# Supplementary figures and images for: Benefits and unintended consequences of antimicrobial de-escalation: Implications for stewardship programs
Source: PLoS One. 2017 Feb 9;12(2):e0171218. doi: 10.1371/journal.pone.0171218 (PMC5300270; doi:10.1371/journal.pone.0171218)

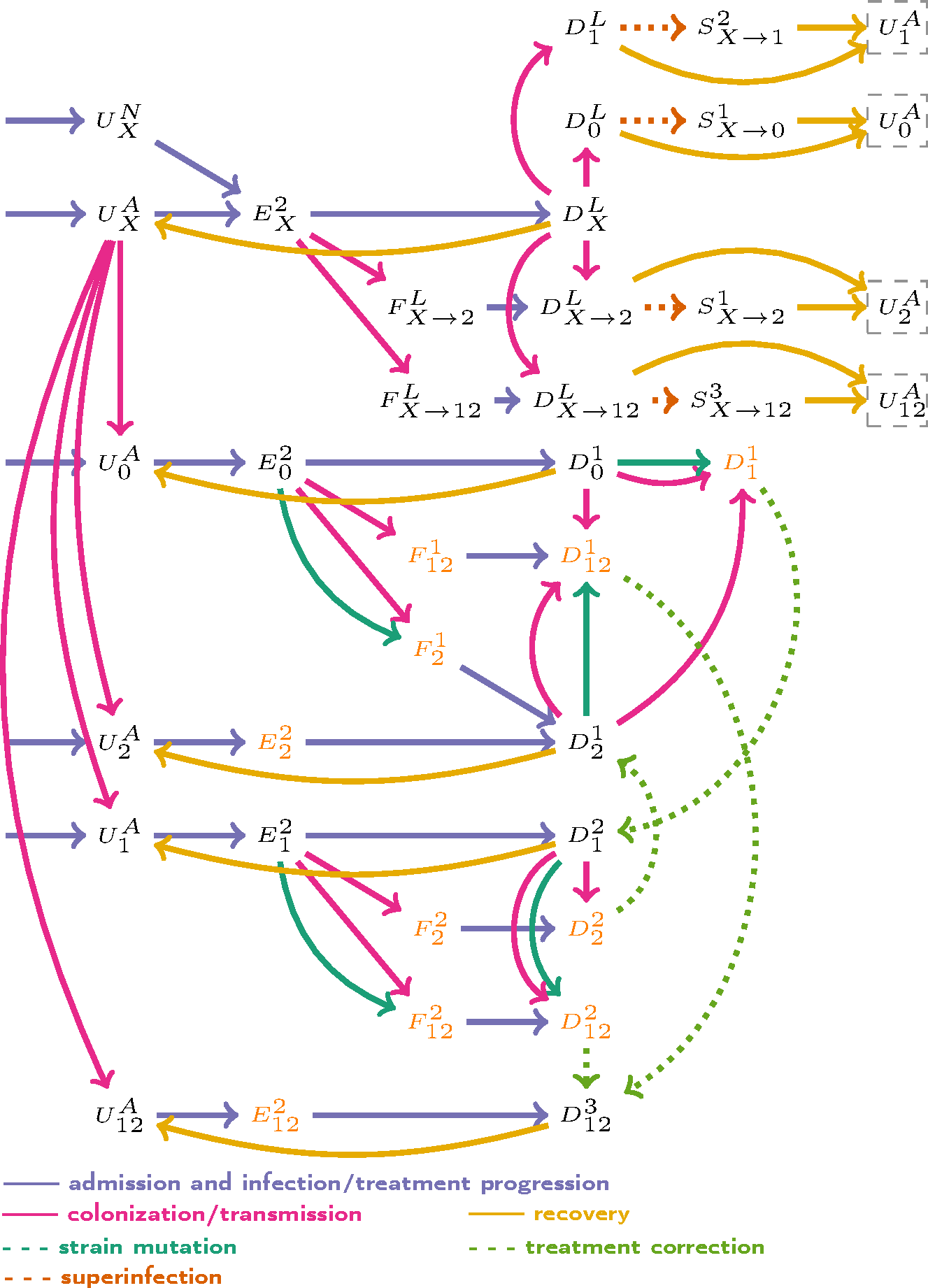

Supplement: S1 Fig — Compartments of patients under inadequate drug treatment are colored in orange. Discharge and death processes are not included in the diagram. See S1 Appendix for notation and equations. (TIF) [file pone.0171218.s002.tif]

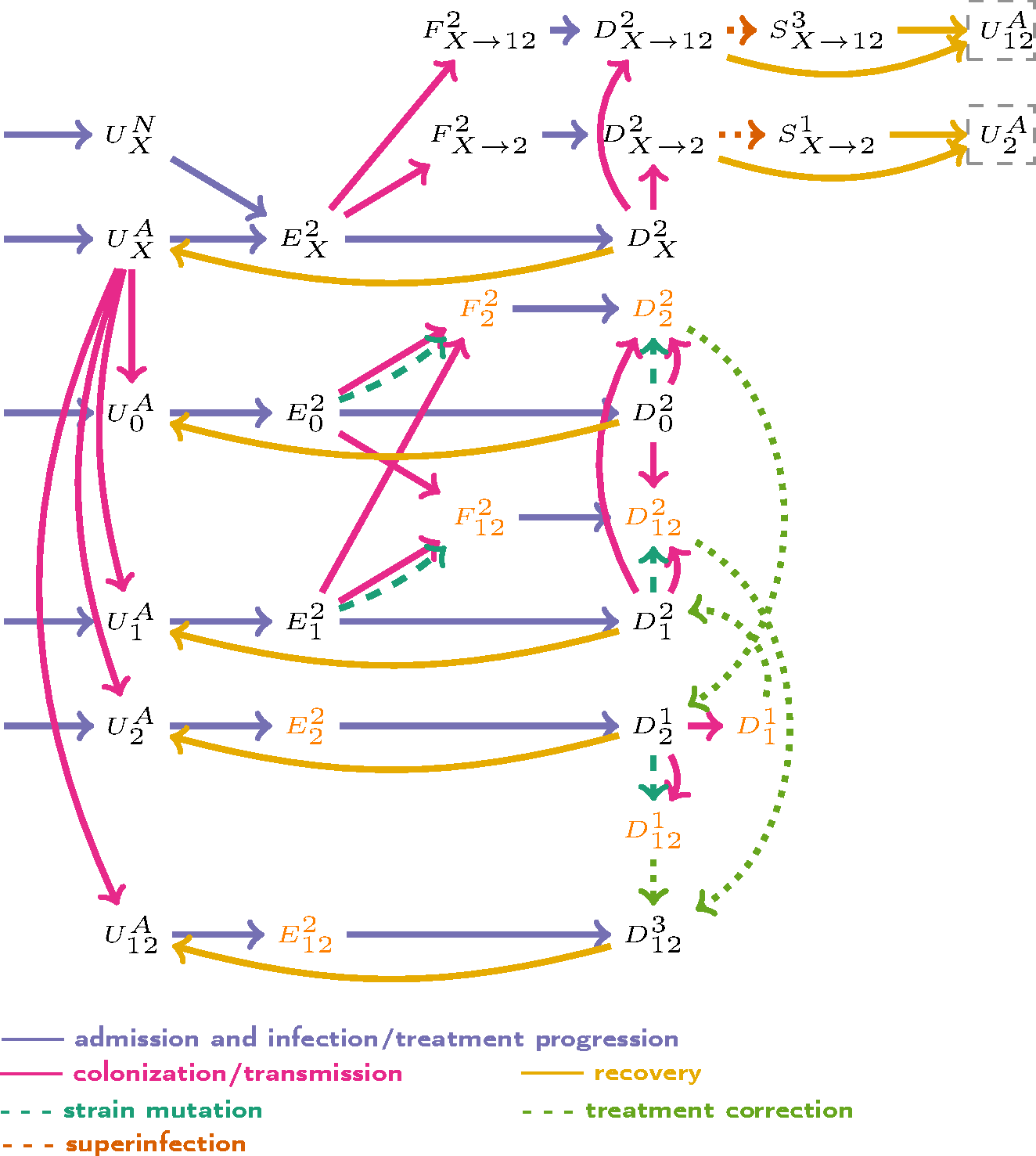

Supplement: S2 Fig — Compartments of patients under inadequate drug treatment are colored in orange. Discharge and death processes are not included in the diagram. See S1 Appendix for notation and equations. (TIF) [file pone.0171218.s003.tif]

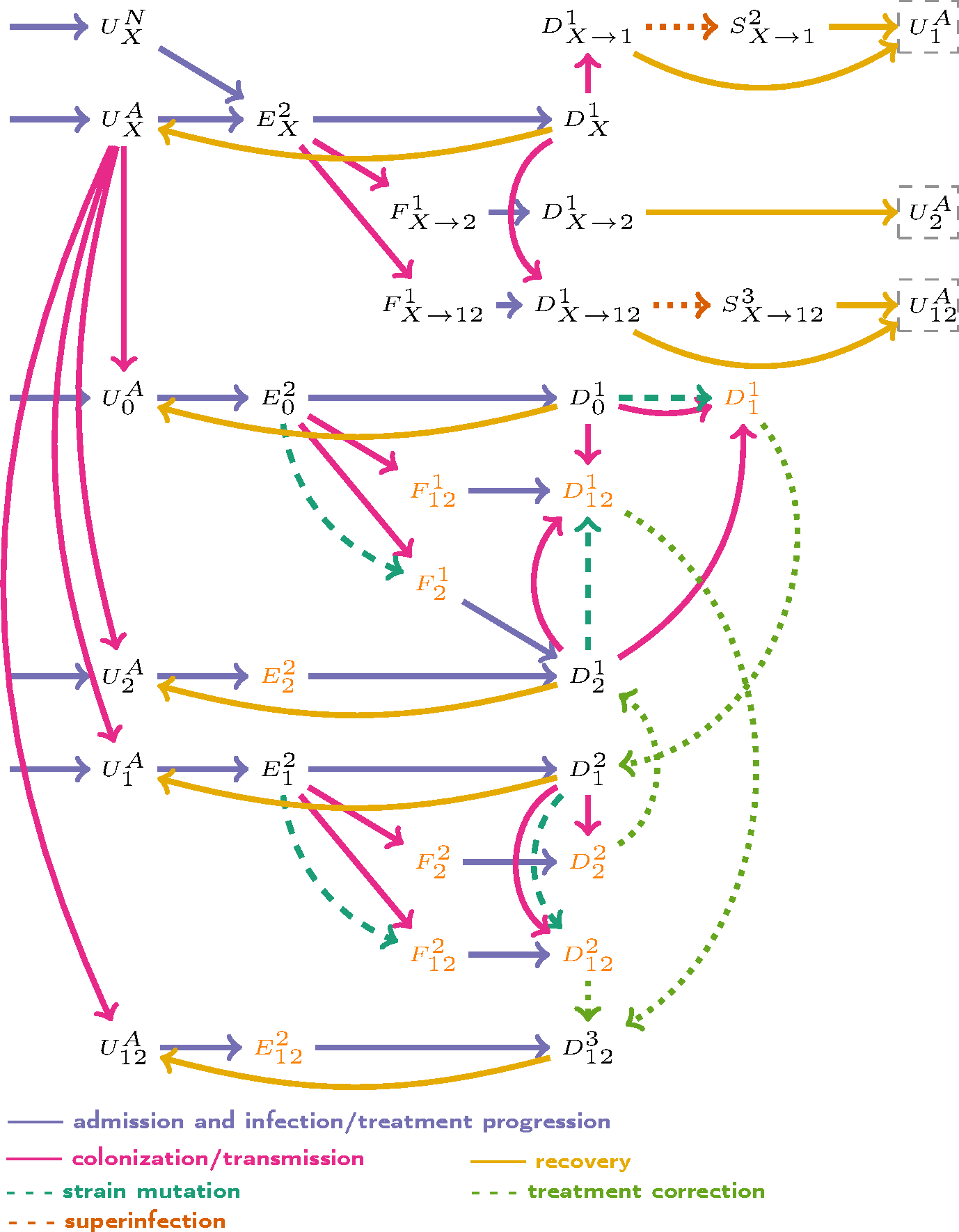

Supplement: S3 Fig — Compartments of patients under inadequate drug treatment are colored in orange. Discharge and death processes are not included in the diagram. See S1 Appendix for notation and equations. (TIF) [file pone.0171218.s004.tif]

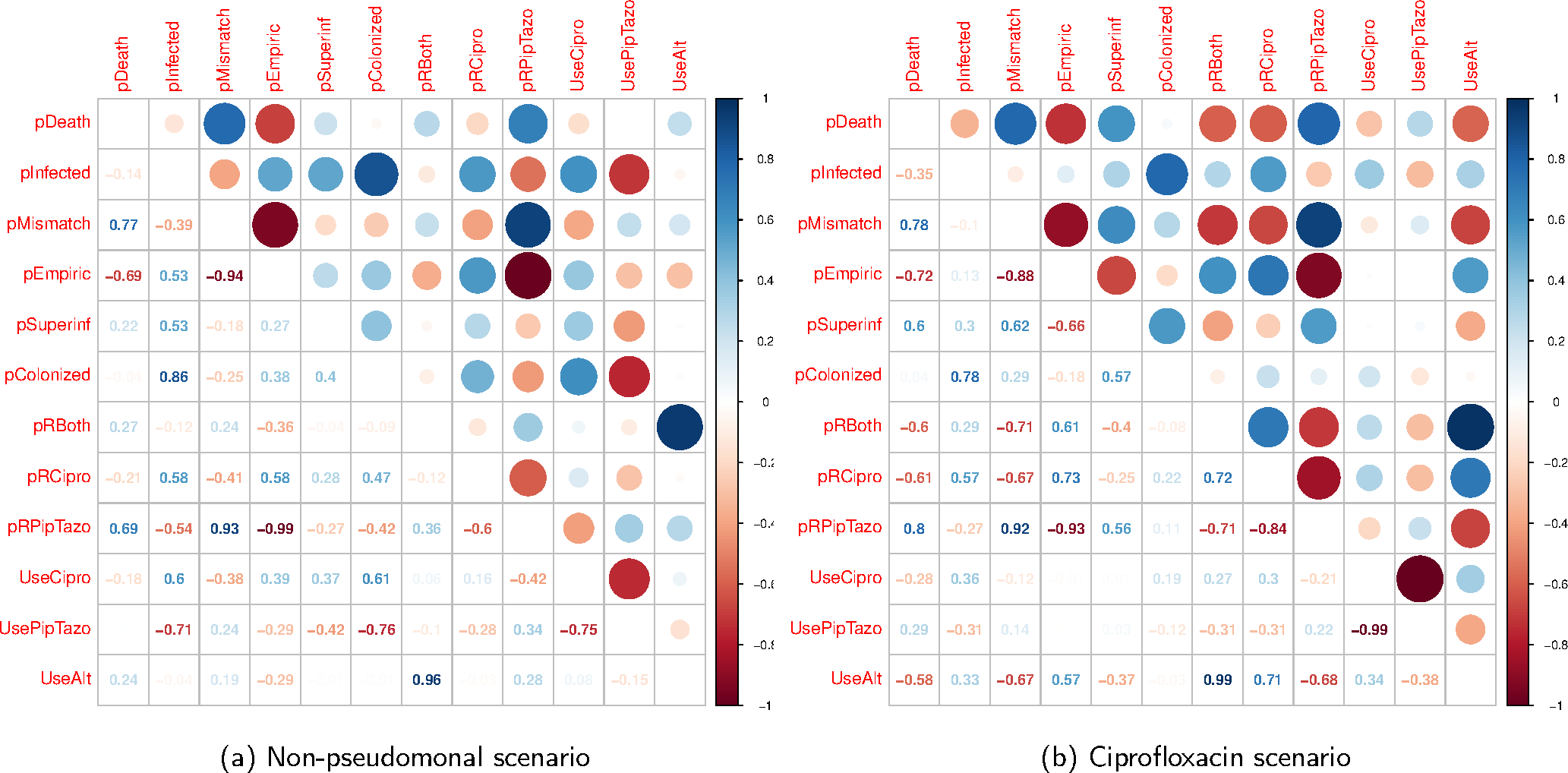

Supplement: S4 Fig — (TIF) [file pone.0171218.s005.tif]

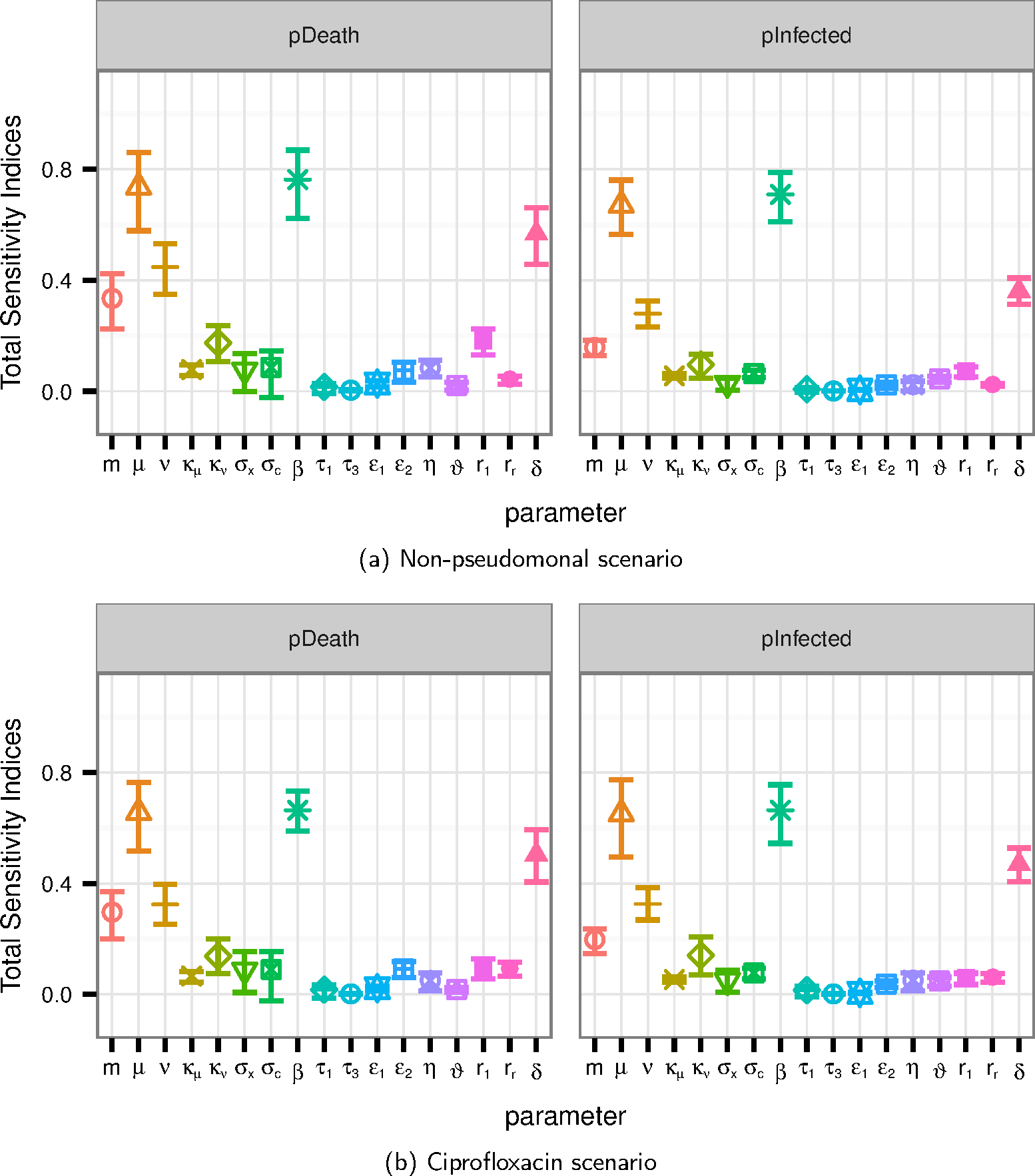

Supplement: S5 Fig — Results include parameter values that do not meet the calibration criteria. (TIF) [file pone.0171218.s006.tif]

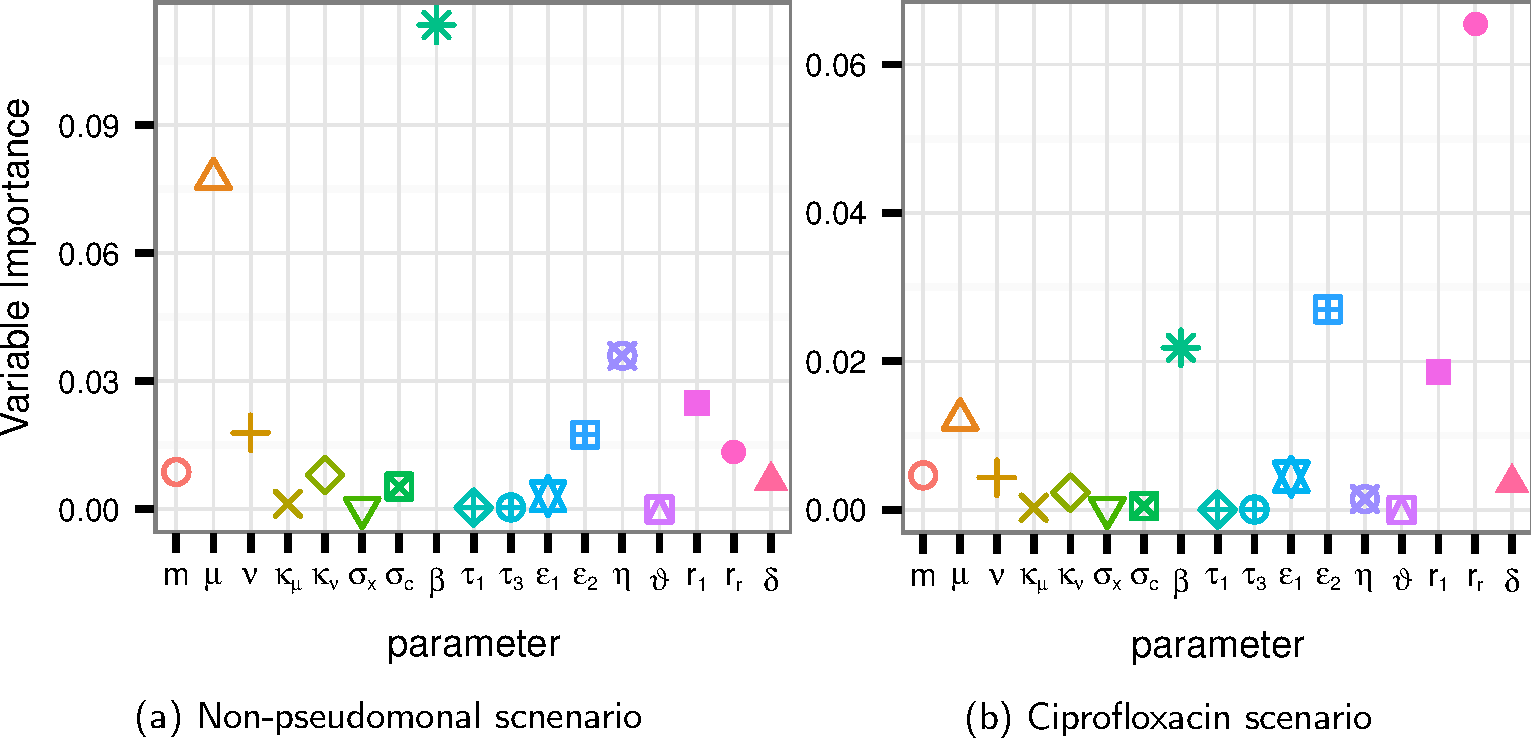

Supplement: S6 Fig — 500 trees gave OOB classification error rates of 13% and 7.5% for the non-pseudomonal and ciprofloxacin scenarios, respectively. (TIF) [file pone.0171218.s007.tif]
